# Interactions Between Killer Whales ( *Orcinus orca* ) and Neonate Long‐Finned Pilot Whales ( *Globicephala melas* ) off South Iceland

**DOI:** 10.1002/ece3.71193

**Published:** 2025-04-23

**Authors:** Chérine D. Baumgartner, Anna Selbmann, Heleen Middel, Josephine Schulze, Giulia Bellon, Filipa I. P. Samarra

**Affiliations:** ^1^ Department of Environmental Systems Science Swiss Federal Institute of Technology Zurich Switzerland; ^2^ Orcestra Zurich Switzerland; ^3^ Faculty of Life and Environmental Sciences University of Iceland Reykjavík Iceland; ^4^ Westman Islands Research Centre University of Iceland Vestmannaeyjar Iceland; ^5^ Center for Geophysical Forecasting, Department of Electronic Systems Norwegian University for Science and Technology Trondheim Norway; ^6^ Leibniz Institute for Baltic Sea Research Warnemünde Rostock Germany

**Keywords:** affiliative, agonistic, interspecific interactions, mixed‐species group

## Abstract

Cetacean interactions can be diverse and complex, spanning agonistic to affiliative behaviors. While killer whales are known for complex hunting strategies and a wide range of prey worldwide, they also engage in various non‐predatory interspecific interactions. This study describes two encounters off south Iceland, on the 23rd of June 2022 and the 20th of June 2023, where neonate pilot whales were observed with killer whale groups. No other pilot whales were sighted in the area for the duration of these encounters. The killer whale groups, of mixed age and sex classes, engaged in slow travel, foraging and social behaviors throughout the encounters. The pilot whale calves were seen surfacing in front of killer whales or in echelon position, and at times being lifted out of the water. Photo‐identification revealed that, while different individual whales participated in each event, all were likely fish‐eating specialists. The interactions with the calves displayed no clear signs of predation. We explore several hypotheses for the function of these interactions, which appear suggestive of play behavior (whether affiliative or agonistic in nature), or possibly practice hunting, or epimeletic behavior. However, because neither interaction between killer whales and a pilot whale neonate was observed from start to end, the true motivation and function of the interactions remain unknown. Our observations not only highlight the complexity of these interactions but also underscore the broader importance of investigating interspecific encounters among cetaceans and other mammals, where such behaviors can offer insights into ecological and social processes.

## Graphical abstract

This study documents two encounters off south Iceland where neonate pilot whales were observed in the company of killer whale groups, with no other pilot whales in the vicinity. The interactions involving the calves showed no clear signs of predation. We explore several hypotheses for the function of these interactions, which appear suggestive of play behavior, or possibly practice hunting, or epimeletic behavior.

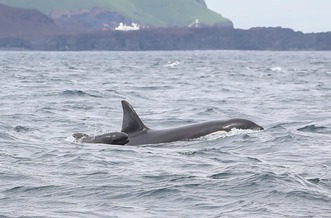

Interspecific interactions among cetaceans can be complex and diverse. Such interactions include predation (e.g., Jefferson et al. 1991), and non‐predatory interactions ranging from agonistic to affiliative behaviors; such as aggression (e.g., Barnett et al. 2009; Herzing and Johnson 1997; Orr and Harwood 1998; Palacios and Mate 1996; Ross and Wilson 1996; Shane 1995; Shelden et al. 1995; Weller et al. 1996), mating (e.g., Acevedo‐Gutiérrez et al. 2005; Herzing and Johnson 1997), mixed‐species group traveling and playing (e.g., Herzing and Johnson 1997), and epimeletic behavior (Bearzi and Reggente 2018; Baerzi et al. 2018). Observations of mixed‐species groups of cetaceans at sea are not uncommon, with these groups hypothesized to reduce predation risk and increase foraging or social benefits (Syme et al. 2021).

Killer whales ( *Orcinus orca* ) are the ocean's apex predators and their prey includes a wide range of cetacean species (Forney and Wade 2007; Jefferson et al. 1991). Although most killer whale interactions with other marine mammal species are of a predatory nature, non‐predatory interactions have also been regularly documented (Jefferson et al. 1991). These include the formation of mixed‐species groups, instances of close proximity without noticeable reactions from either species, concurrent feeding, and both avoidance of and attraction to killer whale groups (Jefferson et al. 1991). For example, humpback whales ( *Megaptera novaeangliae* ) have been observed in feeding aggregations with killer whales, where humpback whales seem to feed on fish schools that were initially tightly herded by killer whales (Jourdain and Vongraven 2017). On other occasions, and in other parts of the world, humpback whales have been observed interfering with predatory attacks of killer whales on other marine mammals (Pitman et al. 2014). Fish‐eating killer whales have also been observed harassing and killing porpoises ( *Phocoena phocoena* and *Phocoenoides dalli* ) without consuming them (Giles et al. 2023), exemplifying that a fish‐specialist population can engage in lethal, non‐predatory interactions.

Long‐finned pilot whales ( *Globicephala melas* , hereafter pilot whales) are known to interact with killer whales in the North Atlantic (de Stephanis et al. 2014; Stenersen and Similä 2004) and such interactions have recently been documented in Iceland (Selbmann et al. 2022). In these seemingly agonistic, non‐predatory interactions, pilot whales approach killer whales, usually at high speed. The killer whales avoid the pilot whales, sometimes swimming away at high speed (porpoising). The pilot whales were never observed reaching the killer whales or engaging in close or physical contact (Selbmann et al. 2022). These interactions may be driven by competition for resources or a mobbing anti‐predator strategy, but the exact causes remain unknown (Curé et al. 2012, 2019; de Stephanis et al. 2014; Selbmann et al. 2022; Stenersen and Similä 2004).

In Iceland, herring ( *Clupea harengus* ) is the killer whales' main food source, however, the feeding ecology of the population is complex. A subset of Icelandic herring‐feeding killer whales is known to have a mixed diet including both fish and marine mammals (Samarra and Foote 2015; Samarra et al. 2017a). Predation events on marine mammals recorded in Iceland (by known and by unidentified individuals) include both pinnipeds and cetaceans (Samarra et al. 2018), although it is unknown if there are any killer whales that feed exclusively on marine mammals. Only one potential predation on pilot whales has been reported in Iceland (Donovan and Gunnlaugsson 1989), but killer whales are known to chase, attack (Bloch and Lockyer 1988; García‐Godos 2004), or predate on both long‐ and short‐finned pilot whales ( *Globicephala macrorhynchus* ) elsewhere (Heide‐Jorgensen 1988; Jefferson et al. 1991; Nishiwaki and Handa 1958). Some of these observations were made in nearby regions to Iceland, such as the Faroe Islands and southwest Greenland (Bloch and Lockyer 1988; Heide‐Jorgensen 1988).

In 2021, a pilot whale neonate was observed with a small group of killer whales in the west of Iceland (Mrusczok et al. 2023), with unknown origin or outcome of the event (i.e., predatory vs. non‐predatory). During this 21‐min observation, the pilot whale calf was observed surfacing in echelon position next to a female killer whale. Here, we describe two additional accounts where neonate pilot whales were observed with groups of killer whales in south Iceland, and we discuss the potential functions of these interactions.

On the 23rd of June 2022 at 10:20 UTC, a killer whale group was sighted by observers conducting visual surveys from a land station, a vantage point at an altitude of 100 m at the southern tip of Heimaey island (Figure 1). The observers communicated the sighting to the research vessel team, who reached the killer whale group at 11:15 UTC. The boat‐based observers noted the presence of a neonate pilot whale, of very small size and with visible fetal folds, within the killer whale group. The observers recorded photo‐identification, surface behavior, video and acoustic recordings. Photo‐identification was collected with a Canon EOS70D camera equipped with a Canon EF70‐200 mm f/2.8 L IS II USM lens. Individual killer whales were identified by the shape and size of the dorsal fin and saddle patch, as well as nicks on the dorsal fin and scars on the saddle patch (Bigg 1982) and compared to an existing photo‐ID catalog (Samarra et al. 2017c). Surface behavioral observations were recorded as voice notes and/or on a data sheet *ad libitum*, but at least every 5 minutes. Observations included the documentation of group size and composition, surface behaviors such as tail slaps and logging, swimming direction, indicators of feeding, such as scales or prey remains, and prey species. Videos were collected opportunistically with mobile phones (Apple iPhone 11pro). Acoustic recordings were collected using a single hydrophone (HTI‐96‐MIN Marine Mammal) at 7 m depth deployed from the boat, recording at 192 kHz sampling rate onto a Tascam X8 Recorder. The recordings were analyzed aurally and visually using spectrograms (Hann window, FFT = 8192) generated in Audacity (Audacity Team 2014, version 3.2.3).

**FIGURE 1:**
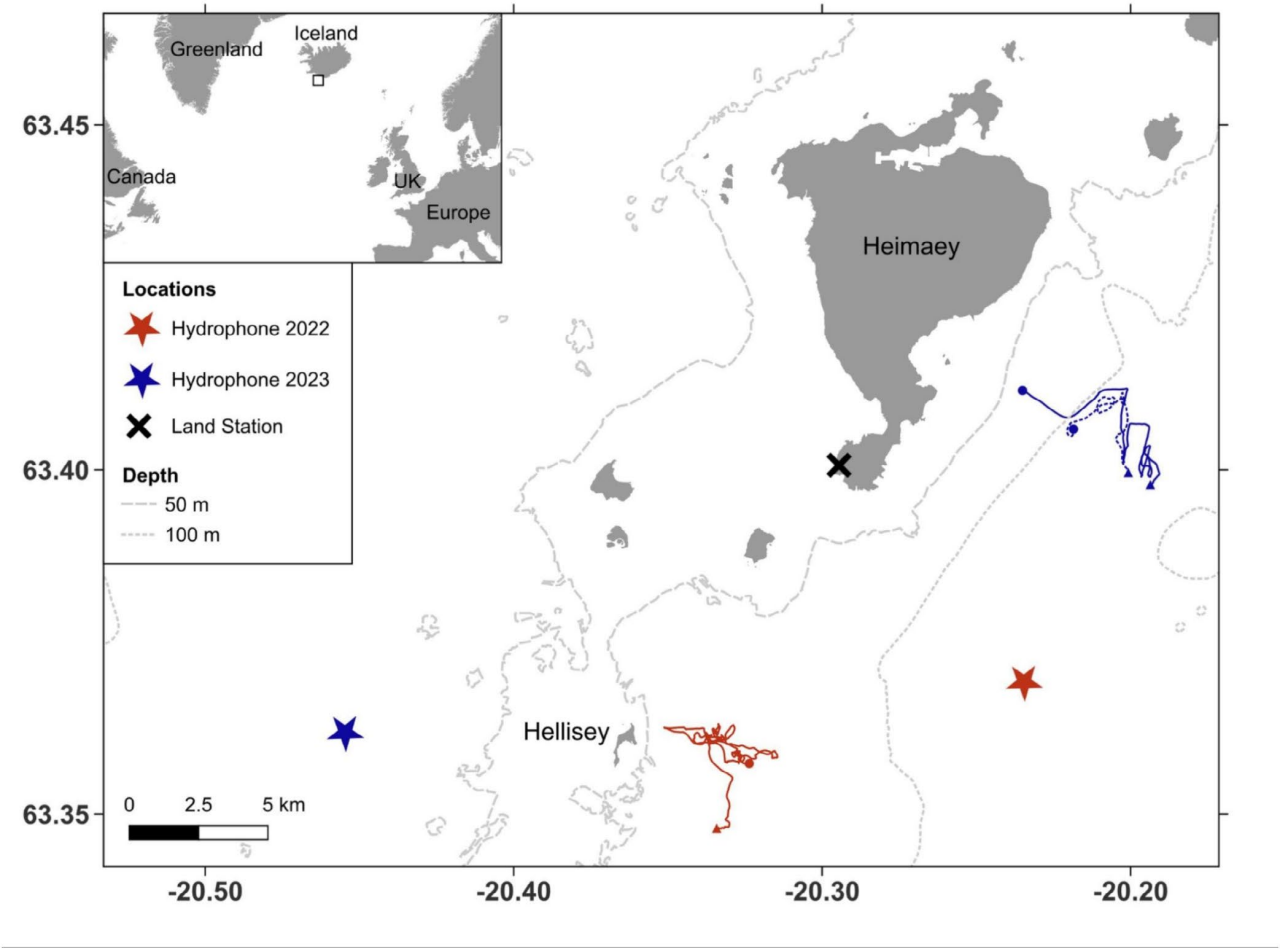
Map indicating the encounters of neonate long‐finned pilot whales swimming with killer whales in Vestmannaeyjar (Iceland) in 2022 and 2023. The track of the research vessel during the encounters is given for June 23, 2022 (red) and June 20, 2023 (blue). An additional dotted blue line shows the vessel's track during an encounter with the same group of killer whales on June 21, 2023. The starts and ends of tracks are indicated with dots and triangles, respectively. Observers registered sightings from a land station on Heimaey island (black cross). The locations of bottom‐moored hydrophones used in 2022 (red star) and 2023 (blue star) are also indicated. The inset map shows the location within the North Atlantic. The map was generated using QGIS (QGIS Development Team 2022), with data on land limits from the IS 50 V database of the National Land Survey of Iceland (2022).

The group was composed of 11 killer whales: six adults (males IS033, IS268, and IS413, and females IS032, IS366, and IS379) and five juveniles (IS512, IS526, IS570, and two unidentified juveniles; Samarra, unpublished data). All identified individuals had been previously sighted in this region in multiple years. All adult whales, except IS268 and IS379, are known to occur in both herring winter and summer spawning grounds and form part of a known subgroup of cluster E (Tavares et al. 2017). Based on their movement patterns, the adult whales sighted in both herring winter and spawning grounds are likely fish‐specialists, following the herring migration year‐round (Samarra et al. 2017b). Although chemical tracer information on diet was not available for any of the identified whales, earlier analyses showed that those whales following herring year‐round exhibited lower δ15N values and a significantly narrower trophic niche width, consistent with a fish‐specialist diet (Samarra et al. 2017a). Furthermore, killer whales with a diet including marine mammals have higher contaminant levels (Remili et al. 2021) and a distinct fatty acid profile (Remili et al. 2023) compared to sympatric fish‐eating specialists. Throughout the nearly 3 h duration of the encounter, killer whales changed behavior between feeding, most likely on herring, and apparent socializing. Overall, no consistent direction of movement was observed. In both behavioral states, surface‐active behavior was common. In particular, two juveniles were active at the surface; the observers recorded several breaches, twirls just beneath the surface, splashing, spy hopping, body contact, and tail slaps on the surface. The killer whales appeared to surface on both sides of the pilot whale neonate occasionally (Figure 2) and the pilot whale neonate also surfaced in echelon position relative to a killer whale a few times (Figure 3).

Relevant video footage where the pilot whale calf was observed or showing the killer whale group behavior was compiled into one file of 02:34 min duration (see Video S1). The pilot whale calf is seen in 10 instances, 8 of which show the calf surfacing slightly before a killer whale appears at the surface. In these sequences, the killer whale is positioned right behind and with its head facing towards the pilot whale calf (time stamps in Video S1: 00:02, 00:10, 00:19, 01:19, 01:22, 01:27, 01:38, 02:04). The killer whale's head appears to be very close to the pilot whale calf and might even touch its body, seemingly nudging the calf towards the surface with the pilot whale calf appearing to respond often with tail slapping. However, it could not be clearly determined if this was an affiliative or agonistic behavior. When the calf was not visible at the surface, killer whale behavior occasionally included surface active behavior with milling and sporadic tail slaps (01:00 to 01:26, time stamp 01:49 and 02:25 to 02:34). Milling is commonly associated with foraging upon herring.

**FIGURE 2:**
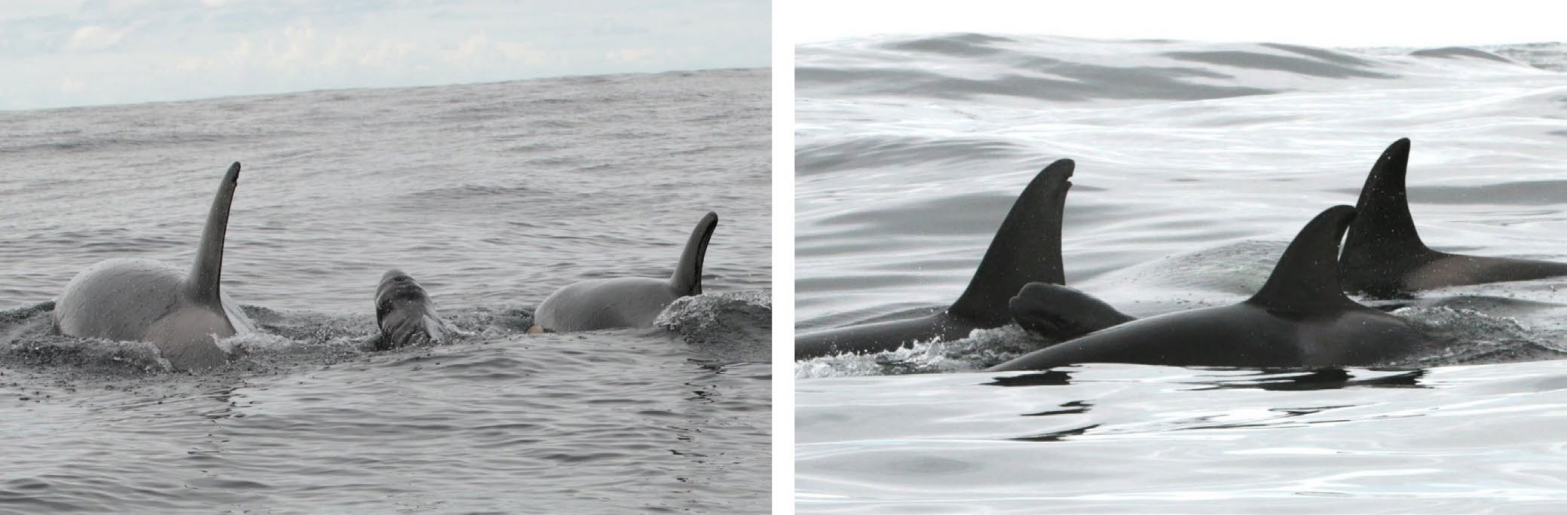
A neonate long‐finned pilot whale swims among killer whales on June 23, 2022. Photos by Josephine Schulze.

**FIGURE 3:**
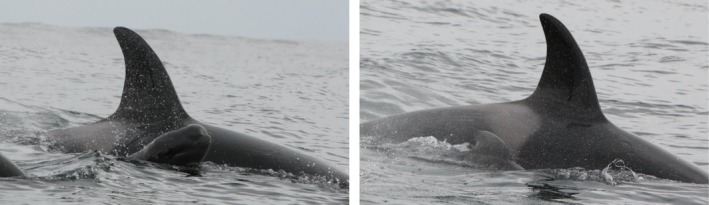
A neonate long‐finned pilot whale surfaces in echelon position next to a killer whale on June 23, 2022. Photos by Josephine Schulze.

The acoustic recordings (total duration 36.5 min) contained frequent echolocation clicks, pulsed calls, and whistles. The detection of two underwater tail slaps supported the visual observations that the killer whales were foraging, likely upon herring (Samarra and Miller 2015; Simon et al. 2005; Similä and Ugarte 1993). No pilot whale vocalizations or “uncommon” sounds were detected in the recordings that could have been assigned to the pilot whale calf.

The observations ended at 14:05 UTC as the research vessel left the whales to return to the harbor. At 15:42 UTC, the land‐based observers also terminated their efforts due to incoming rain and low visibility. No other pilot whales were sighted in the study area on the day of this encounter from the research vessel or land station. Throughout 2022, a total of 43 days of observational effort were conducted—comprising 15 days in June, 16 days in July, and 12  days in August—utilizing both land station and boat‐based surveys. Sightings of pilot whales in the area only occurred on the 6th of June, 13th of June, and 23rd of July 2022. This extensive survey effort enhances the likelihood that the absence of sightings on other days reflects the true distribution of pilot whales rather than missed detections, although the impact of adverse weather conditions on detection probability cannot be entirely ruled out. No pilot whale sounds were detected on a moored hydrophone (Soundtrap ST500 recording at 96 kHz sampling rate with a duty cycle of 44 min on and 16 min off; Figure 1) in the 24 h before or after the observation. The pilot whale calf was not re‐sighted throughout the remainder of the field season (last day of field effort was the 27th August 2022) despite some individuals from this group of killer whales being sighted on the 27th of June 2022.

On the 20th of June 2023 at 13:46 UTC, a group of killer whales was sighted on the east side of Heimæy. During the approach of the research vessel, breaching, tail slaps and repeated splashes were seen from a distance. Upon arrival, the boat‐based observers noted the presence of a neonate pilot whale, of very small size and with visible fetal folds, with the killer whale group. Similar to the previous encounter, the observers collected photo‐identification, behavior, and surface observations. Photo‐identification was collected with a Canon EOS5D Mark IV camera equipped with a Canon EF70‐300 mm f/4–5.6 L IS USM lens. The group was composed of eight killer whales: three adult males (IS008, IS040, IS046), two adult females (IS069, IS270), one “other” (i.e., an adult sized individual but of unknown sex, IS397), and two juveniles (IS569 and an unidentified juvenile). All identified individuals belonged to a well‐known subgroup of cluster I (Tavares et al. 2017) and had been previously sighted multiple times and in multiple years in this region. In our database, we only have records of these individuals at herring spawning grounds. Individuals IS008, IS046, and IS069 are classified as fish‐specialists, based on their lower nitrogen isotope (δ15N) and lower contaminant loads compared to killer whales that incorporate both fish and mammals in their diet, both of which are consistent with a fish‐specialized diet (Samarra et al. 2017a; Remili et al. 2021). Thus, it is likely that all individuals in this group are fish‐specialists.

The group was slowly traveling back and forth in the same area, maintaining a tight group composition. The pilot whale calf was repeatedly seen swimming in echelon position relative to adult female IS069 throughout the encounter (Figure 4). This behavior was observed until 14:13 UTC, when the group started to speed up. Multiple surfacings revealed erratic movement patterns among the killer whales: they would travel briefly in one direction before diving and resurfacing elsewhere (as reflected in the boat track in Figure 1). The pilot whale calf was sighted a few meters ahead of and moving away from the killer whales, indicating possible evasive behavior. At 14:34 UTC, the pilot whale calf was observed being lifted out of the water by an adult killer whale, possibly adult female IS069, although the ID could not be confirmed (Figure 5). Unfortunately, deteriorating weather conditions did not allow observers to continue behavioral observations and by 14:36 UTC (after approximately 1 h) the boat had to return to the harbor.

**FIGURE 4:**
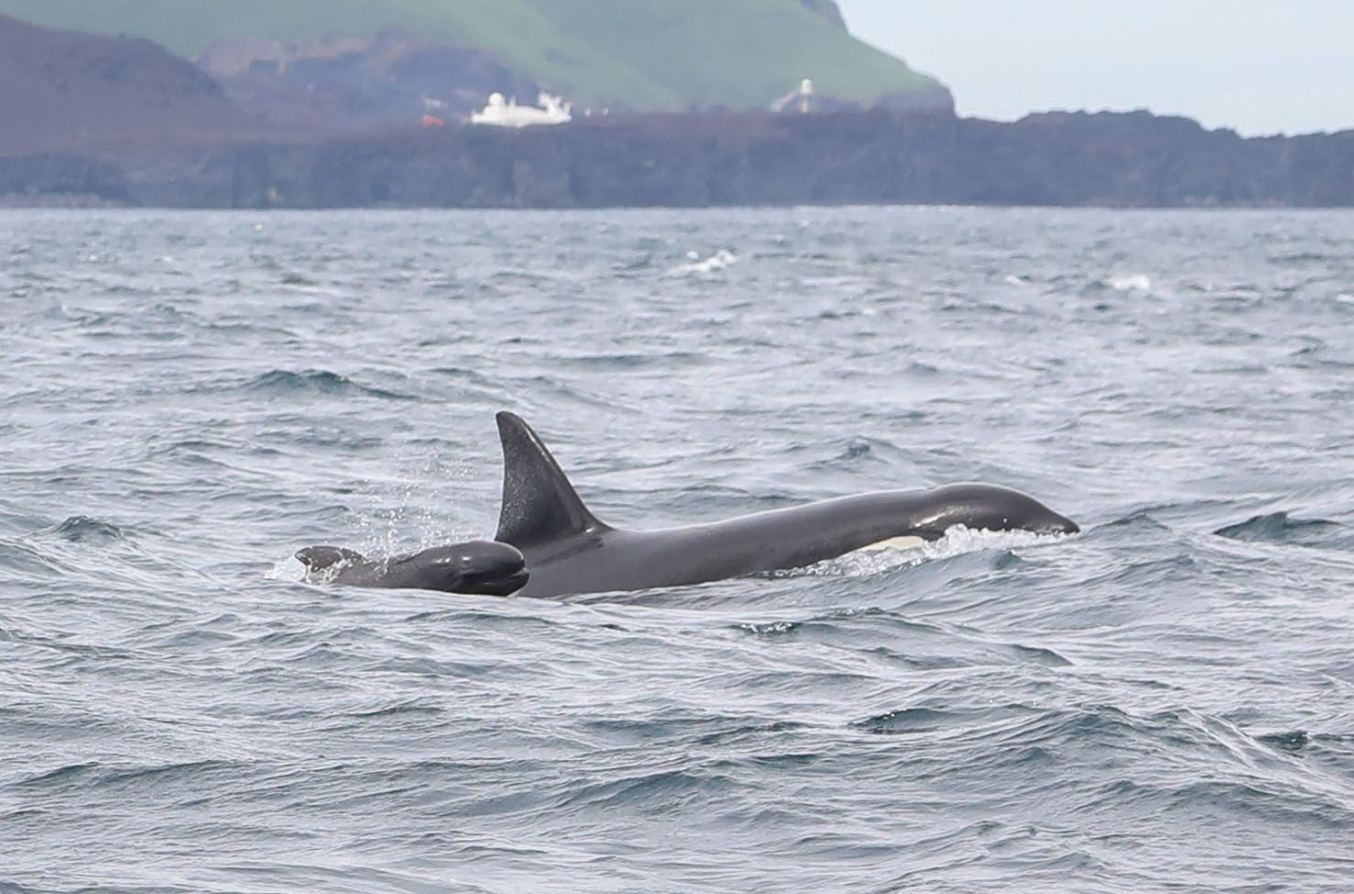
A neonate long‐finned pilot whale surfaces in echelon position next to a killer whale (adult female IS069) on June 20, 2023. Photo by Filipa Samarra.

**FIGURE 5:**
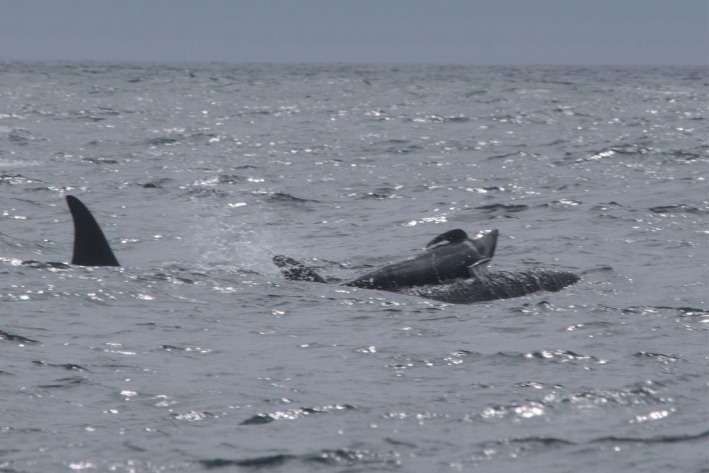
A neonate long‐finned pilot whale is lifted out of the water on the back of an unidentified killer whale on June 20, 2023. Photo by Heleen Middel.

The following morning (June 21, 2023), the same killer whale group was encountered again at the same location, with all individuals confirmed by photo‐identification except the unidentified juvenile (Figure 1). The group was again slowly traveling back and forth in a small area. Observers onboard the research vessel collected information on the animals for approximately 38 min, after which the sea state forced the team to leave. The pilot whale calf was not observed with the group, and because initial sea conditions were similar to the day before, we are confident it would have been detected if still present. The calf was not re‐sighted during the remainder of the field season (last field effort day: August 25, 2023).

No other pilot whales were sighted on the day of the encounter from the research vessel or land station. In 2023, a total of 38 days of effort were undertaken—13 days in June, 16 days in July, and 9 days in August—employing both land station and boat‐based surveys. Pilot whales were sighted on June 21, 2023 and on several days in July (3rd, 15–17th, 19th, 20th, 22nd, 24th, 25th). Pilot whale sounds were detected by a bottom‐moored hydrophone (Soundtrap ST600HF recording continuously at 192 kHz sampling rate; Figure 1) on the day of the interaction from 03:52 to 11:17 UTC. This corresponds to up to about 2.5 h before the observation of the interaction began. Pilot whale sounds were detected again from 21:40 UTC onwards, that is approximately 7 h after the observation ended. In contrast, killer whale sounds were detected almost continuously throughout the 24 h before and after the observation.

In this study, we report two accounts of neonate pilot whales observed with groups of killer whales in south Iceland. There are several potential motivations and functions for the interactions we report here, which we discuss below.

The proximity of neonate pilot whale calves to killer whales could suggest predatory behavior. Killer whales predating on pilot whales have been documented in nearby regions (Heide‐Jorgensen 1988), although only one potential predation event has been recorded in Iceland to date, approximately 58 km east of Heimaey island (Donovan and Gunnlaugsson 1989). Yet, the majority of the killer whales involved in the interactions we report are likely fish‐specialists, based on movement patterns and/or chemical tracers (Samarra et al. 2017a, 2017b; Remili et al. 2021). Additionally, the spatio‐temporal occurrence of killer whales in this region is associated with high herring abundance, and concurrent foraging on herring was recorded during one of the interaction events. Occasional predation on marine mammals cannot be ruled out for all killer whales involved in our observations. However, the fact that at least some of the whales involved are known fish‐specialists, along with the absence of overtly aggressive behavior or significant injury in the pilot whale calf, support the hypothesis that these were non‐predatory interactions.

Non‐predatory interactions are common among delphinids, encompassing a range of behaviors from affiliative to agonistic, with varying levels of aggression. While some studies have documented high levels of aggression (Barnett et al. 2009; Cotter et al. 2012; Crespo‐Picazo et al. 2021; Giles et al. 2023; Wedekin et al. 2004), in other occasions physically dominant species refrain from inflicting the full extent of harm they are capable of (Baird 1998; Haelters and Everaarts 2011), and some interactions are affiliative (Acevedo‐Gutiérrez et al. 2005; Bearzi and Reggente 2018; Baerzi et al. 2018). Interactions between delphinids and neonates or calves of other species have been observed; however, the initial context and ultimate outcomes of these events often remain unclear, and the observed behaviors do not allow for a single, definitive interpretation (Baird 1998; Giles et al. 2023; Haelters and Everaarts 2011; Mrusczok et al. 2023). Recently a large data set of a non‐predatory behavior in a fish‐specialist killer whale population has been analyzed: Southern Resident killer whales in the North Pacific engage in non‐predatory interactions, which involve harassment and killing of porpoises (Giles et al. 2023). During these interactions, killer whales exhibit low and high activity behaviors, including chasing and herding the porpoise, tossing it into the air, cradling or squeezing between individuals, lifting or carrying the porpoise on the killer whale's back, head, or rostrum, and swimming in echelon position (Giles et al. 2023). Given the involvement of both sexes and all age classes, the authors concluded that several hypotheses for this behavior were plausible, including a form of play with social or developmental benefits, practice hunting, and/or displaced epimeletic behavior for reproductive females (Giles et al. 2023).

Interactions with pilot whale calves off the coast of Iceland show certain characteristics that could be interpreted as play—characterized as a spontaneous and pleasurable behavior with no relevance for survival (Burghardt 2005), which is typically, though not exclusively, associated with young individuals (see Kuczaj and Eskelinen 2014). However, it remains unclear whether our behavioral observations deviate from typical juvenile activity in killer whales. And, although the heightened surface activity noted in the first event and the very beginning of the second event may suggest playful elements, the second event was largely dominated by adult interactions and traveling behavior. Moreover, even if certain aspects of these interactions appear “playful”, this does not necessarily indicate that play was the primary motivator for these interactions. Furthermore, definitions of play often exclude foraging contexts (Burghardt 2005), indicating the possibility for alternative interpretations and/or multiple explanations for the behaviors observed.

An interrelated behavior to play is practice hunting, which may offer benefits for group cohesion and coordination (Giles et al. 2023). Killer whales have been documented engaging in practice hunting that involves both adults and juveniles, indicating potential teaching and skill development within groups (e.g., Guinet 1991). Off the coast of Iceland, killer whales herd herring into tight bait balls as part of their foraging technique (Samarra and Miller 2015; Simon et al. 2007), similar to the carousel feeding behavior observed in herring‐eating killer whales off Norway (Similä and Ugarte 1993). Observations of a female and a juvenile swimming on either side of the pilot whale calf may indicate training exercises aimed at preventing prey from evading. Such coordinated behavior could serve as practice for herding techniques used during actual feeding, though this remains untested. Benefits for group coordination and cohesion have been proposed as hypotheses for similar behaviors in common bottlenose dolphins ( *Tursiops truncatus* ), which have been observed coordinating lethal, non‐consumptive attacks on harbor porpoises (Cotter et al. 2012).

Another interpretation of our observations is that the killer whales exhibited displaced epimeletic behavior or alloparental care towards the pilot whale calf. Indeed, epimeletic and possible alloparental behavior were suggested as a likely explanation for a brief (21 min) interaction observed in west Iceland in 2021, because the pilot whale calf was often observed swimming in echelon position next to an adult female killer whale (Mrusczok et al. 2023). During our longer encounters (2 h and 50 min duration in the first encounter and 50 min duration in the second), we observed behaviors that could be interpreted as caregiving, such as pushing the pilot whale calf from underneath—potentially to keep it afloat (Bearzi et al. 2018; Bearzi and Reggente 2018)—and the calf swimming in echelon position relative to a nearby killer whale, including adult females. While hydrodynamic advantages are believed to drive echelon swimming in mother–infant pairs (Noren 2008), the same behaviors—pushing from underneath, nudging, and echelon swimming—have also been observed in aggressive contexts (e.g., Baird 1998; Cotter et al. 2012; Giles et al. 2023). In the second encounter, the pilot whale calf sometimes swam ahead of and away from the killer whales, which may be similar to Giles et al. (2023) reporting that Southern Resident killer whales allowed porpoises to escape before recapturing them and resuming behaviors such as pushing, carrying, or balancing the porpoise. However, given that we did not document overt violence, epimeletic behavior and/or alloparental care remain possible motivations for the interactions among other interpretations.

Competition for prey resources between the two species has been proposed as one of the drivers of the agonistic interactions that occur between killer whales and pilot whales (Curé et al. 2012, 2019; de Stephanis et al. 2014; Stenersen and Similä 2004). In observations of interactions in Iceland, killer whales often abandon feeding events to move away from pilot whales (Selbmann et al. 2022). Interactions between killer whales and pilot whale calves may, in this context, represent one aspect of a broader set of interactions potentially related to feeding interference. Our knowledge of the diet of pilot whales in Iceland is limited (Sigurjónsson et al. 1993). Recently, the trophic ecology of pilot whales stranded along the Icelandic coast has been investigated (Samarra et al. 2024), but formal comparisons of trophic niche overlap between killer whales and pilot whales are yet to be conducted (Samarra et al., in preparation). Thus, the potential for competition for prey resources needs to be evaluated before firm conclusions can be drawn. Nevertheless, given that this was not shown to be the cause of interactions between both species in the Strait of Gibraltar (de Stephanis et al. 2014), and that pilot whales are considered predominantly teutophagous, feeding primarily on cephalopods, throughout their range (e.g., Desportes and Mouritsen 1993; Santos et al. 2014; Spitz et al. 2011), this is not a likely driver.

Although several hypotheses discussed above remain plausible, some are more unlikely than others. Given that we did not observe overtly aggressive behaviors, such as those documented in other cetacean interactions (Barnett et al. 2009; Cotter et al. 2012; Crespo‐Picazo et al. 2021; Giles et al. 2023; Towers et al. 2018; Wedekin et al. 2004), we do not consider territorial defense, or practice related to infanticide or to fighting as likely drivers of the behaviors we observed. Similarly, sexual aggression or high testosterone levels in males are unlikely explanations. If that was the case, we would expect males to engage more in such behaviors; however, the pilot whale calves were primarily in close proximity to females and juveniles. Moreover, the involvement of different individuals across the three documented encounters so far (two encounters reported in our study and one in Mrusczok et al. 2023) suggests that this is not an atypical behavior of a single individual.

It is important to recognize that all three encounters reported to date off Iceland were brief and, thus, may each represent only a segment of a longer sequence of events. For example, understanding the origins of these events could provide valuable insight into the causes of these interactions. Mrusczok et al. (2023) suggest some killer whales may approach pilot whale groups to obtain pilot whale calves. They also raise the possibility that lone neonates—possibly abandoned or lost—could be encountered opportunistically by killer whales. Alternatively, lone pilot whale neonates might themselves locate and initiate contact with killer whales. While we have never observed lone pilot whale neonates in the wild, strandings of lone pilot whale neonates in the south of Iceland have been reported: one in 2022 and one in 2024 (Halldórson, personal communication). It is unknown whether these particular strandings involved live or deceased animals. The 2022 stranding occurred near Landeyjahöfn, the closest point on the mainland to our study site, three weeks after we observed a pilot whale neonate with a group of killer whales. Photographic evidence of the stranded calf's dorsal fin and other body parts indicated that it was not the same individual. Such accounts point to a possibility that, for unknown reasons, pilot whale neonates may become naturally separated from their social group, or alternatively, they may wash ashore while the group remains nearby. The circumstances leading up to their stranding remain unclear. To narrow down possible explanations for the observed events, and fully understand them, observing the entire sequence—from the initial separation of pilot whale calves to their eventual release or death—would be necessary.

This study adds to a growing body of literature on poorly understood killer whale and pilot whale interactions and shows that these interactions can be more complex than previously thought. Although pilot whale and killer whale interactions are observed elsewhere (de Stephanis et al. 2014; Stenersen and Similä 2004), encounters of neonate pilot whales with killer whale groups in the absence of any other pilot whales have not been reported outside of Icelandic waters to date. It remains unclear whether this reflects a genuine regional difference or merely that such behavior has not been documented elsewhere to date.

Interpreting behaviors can be difficult and susceptible to observer subjectivity (Mann and Würsig 2014; Nowacek et al. 2016). Notably, in the three events of interactions between pilot whale neonates and killer whales reported to date, the initial context and ultimate outcome remain unknown. Thus, it is difficult at this point to conclude on a single, definitive interpretation of the proximate or ultimate causes of these interactions and it is possible that they have multiple drivers. So far, different killer whale groups have been involved in all three events reported, but we do not know how widespread this behavior is within the population. Future observations of these species interacting should help determine if this is a phenomenon that is spreading, similar to the porpoise harassing and killing by Southern Resident killer whales (Giles et al. 2023), or just isolated, rare and/or opportunistic events. Additionally, adopting a spatial perspective (e.g., through drones when feasible) may provide more clarity on the nature of these interactions and help tease apart their underlying complexities.

## Author Contributions

**Chérine D. Baumgartner:** conceptualization (equal), data curation (lead), investigation (equal), methodology (equal), project administration (equal), visualization (lead), writing – original draft (lead), writing – review and editing (equal). **Anna Selbmann:** data curation (supporting), investigation (equal), methodology (supporting), visualization (supporting), writing – original draft (supporting), writing – review and editing (supporting). **Heleen Middel:** data curation (supporting), investigation (equal), visualization (supporting), writing – original draft (equal), writing – review and editing (supporting). **Josephine Schulze:** data curation (supporting), investigation (supporting), visualization (supporting), writing – original draft (supporting), writing – review and editing (supporting). **Giulia Bellon:** data curation (supporting), investigation (supporting), writing – original draft (supporting), writing – review and editing (supporting). **Filipa I. P. Samarra:** conceptualization (equal), data curation (supporting), funding acquisition (lead), investigation (equal), methodology (equal), project administration (lead), supervision (lead), visualization (supporting), writing – original draft (equal), writing – review and editing (equal).

## Conflicts of Interest

The authors declare no conflicts of interest.

## Supporting information


**Video S1.** Video footage documenting interactions between a neonate long‐finned pilot whale and a group of killer whales in Vestmannaeyjar, Iceland, on June 23, 2022. The 2:34‐min compilation, recorded between 10:20 and 14:05 UTC, captures key sequences of the pilot whale calf near the killer whales, as well as killer whale group behavior.

## Data Availability

All relevant data supporting the findings of this study are included within the manuscript.
